# Diagnostic Value of Dynamics Serum sCD163, sTREM-1, PCT, and CRP in Differentiating Sepsis, Severity Assessment, and Prognostic Prediction

**DOI:** 10.1155/2013/969875

**Published:** 2013-07-01

**Authors:** Longxiang Su, Lin Feng, Qing Song, Hongjun Kang, Xingang Zhang, Zhixin Liang, Yanhong Jia, Dan Feng, Changting Liu, Lixin Xie

**Affiliations:** ^1^Department of Respiratory Medicine, Chinese PLA General Hospital, 28 Fuxing Road, Haidian District, Beijing 100853, China; ^2^Medical College, Nankai University, Tianjin 300071, China; ^3^Nanlou Respiratory Disease Department, Chinese PLA General Hospital, Beijing 100853, China; ^4^Department of Respiratory Medicine, Guangzhou Women and Children Medical Care Center, Guangzhou, Guangdong 510623, China; ^5^Department of Critical Care Medicine, Chinese PLA General Hospital, Beijing 100853, China; ^6^Department of Medical Statistics, Chinese PLA General Hospital, Beijing 100853, China

## Abstract

*Objective*. To describe the dynamics changes of sCD163, soluble serum triggering receptor expressed on myeloid cells-1 (sTREM-1), procalcitonin (PCT), and C-reactive protein (CRP) during the course of sepsis, as well as their outcome prediction. *Patients and Methods*. An SIRS group (30 cases) and a sepsis group (100 cases) were involved in this study. Based on a 28-day survival, the sepsis was further divided into the survivors' and nonsurvivors' groups. Serum sTREM-1, sCD163, PCT, CRP, and WBC counts were tested on days 1, 3, 5, 7, 10, and 14. *Results*. On the ICU admission, the sepsis group displayed higher levels of sTREM-1, sCD163, PCT, and CRP than the SIRS group (*P* < 0.05). Although PCT and sTREM-1 are good markers to identify severity, sTREM-1 is more reliable, which proved to be a risk factor related to sepsis. During a 14-day observation, sCD163, sTREM-1, PCT, and SOFA scores continued to climb among nonsurvivors, while their WBC and CRP went down. Both sCD163 and SOFA scores are risk factors impacting the survival time. *Conclusion*. With regard to sepsis diagnosis and severity, sTREM-1 is more ideal and constitutes a risk factor. sCD163 is of a positive value in dynamic prognostic assessment and may be taken as a survival-impacting risk factor.

## 1. Introduction

Sepsis is one of the most important causes of morbidity and mortality in the intensive care unit (ICU). Multiple organ dysfunction syndrome (MODS) is common among critical cases of severe sepsis and a primary cause of death [1]. Although mortality is rather variable around the world (the rates between 20% and 63%), 750,000 sepsis cases and 210,000 related deaths are reported annually in the United States during the year 2000 [2, 3]. A directory for sepsis diagnosis and treatment, released by the Surviving Sepsis Campaign (SSC) [4, 5], points out that early identification and effective intervention will significantly improve prognosis and reduce death rate [6]. However, current common clinical indicators of infection include pyrexia, white blood cell counts, C-reactive protein (CRP), and procalcitonin (PCT) are still unsatisfactory. Moreover, at present, without timely identification of etiological evidence, nearly 30% of the relevant diagnoses are not well grounded pathologically [7]. Therefore, some of the patients with infection might have their condition worsened, develop multiple organ dysfunction or failure and die for delayed, ineffective treatment [8]. Currently, it is imperative to identify ideal biomarkers capable of making a clear distinction between sepsis and systemic inflammatory response syndrome (SIRS), sepsis severity assessment, and prognostic prediction.

CD163 is the only type of hemoglobin scavenger receptor, specially expressed on the macrophage membrane [9]. Some studies report that the release of inflammatory cytokines caused by the oxidation reduction of hemoglobin plays an important role in the development of severe sepsis [10]. Triggering receptor expressed on myeloid cells-1 (TREM-1) is an immunoglobulin superfamily receptor expressed on polymorphonuclear granulocytes and mature monocytes. Bacteria or fungi infections may upregulate its expression, transmit signals downstream, induce the release of proinflammatory cytokines, and bring about relevant inflammatory responses [11]. PCT test has been put to a wide clinical use because it is a related biomarker, indicating infection and severity [12], as well as prognosis in case of infectious diseases [13, 14]. Although PCT is widely used clinically, its value for sepsis diagnosis has also been challenged recently [12, 15]. CRP is a biomarker involved in more than one inflammatory cascade amplification, now widely applied to sepsis diagnosis [16]. It is also faced with a very awkward situation—CRP proves not to be an ideal biomarker in this field [17, 18]. Therefore, the search for a reliable biomarker for sepsis diagnosis is to continue in the days to come. The present study makes a comparison between four biomarkers (sTREM-1, sCD163, PCT, and CRP) and one scoring system (SOFA scoring system) [19], with the purpose of exploring which of these is/are more valuable in sepsis diagnosis, as well as in the prediction of its development and prognosis. Hopefully, our findings could prove to be of some help to clinicians in general.

## 2. Materials and Methods

### 2.1. Study Subjects

All the subjects were selected from inpatients who were hospitalized between September 2009 and July 2011 in the Respiratory ICU, Surgical ICU, and Emergency ICU, Chinese People's Liberation Army (CPLA) General Hospital. Based on the 2001 American College of Chest Physicians/Society of Critical Care Medicine (ACCP/SCCM) Sepsis Directory [20], patients exhibiting two or more of the following signs during their first 24 h in the ICU were diagnosed as SIRS: (1) temperature of >38°C or <36°C, (2) pulse rate of >90 beats/min, (3) respiratory rate of >20 breaths/min or hyperventilation with a partial pressure of arterial carbon dioxide (PaCO_2_) of <32 mmHg, or (4) white blood cell (WBC) count of >12,000 *μ*L^−1^ or <4000 *μ*L^−1^, or >10% immature cells. Patients exhibiting two or more of SIRS signs with proven infections were to be diagnosed as sepsis. Severe sepsis referred to sepsis complicated by organ dysfunction. Septic shock was defined as a state of acute circulatory failure characterized by persistent arterial hypotension unexplained by other causes. Based on the severity of condition, the sepsis group was further divided into subgroups for sepsis, severe sepsis, and septic shock, respectively. With 28-day survival as the demarcation line, the sepsis patients were also divided into a survivors' group (≥28 days survival) and a nonsurvivors' group (<28 days survival). Patients were excluded if they (1) were younger than 18 years of age; (2) acquired immunodeficiency syndrome; (3) had reduced polymorphonuclear granulocyte counts (<500 *μ*L^−1^); (4) died within 24 h after admission into the ICU, or refused to participate in the study, quit further treatment on their own will during the period of observation. This study was approved by the Ethics Committee of the Chinese People's Liberation Army (CPLA) General Hospital (Projects no. 20090923-001 and no. 20100701-002) and was registered with the U.S. National Institutes of Health Clinical Trials Register (NCT01388725). Patients or their families were fully informed of the details and signed consent forms in this study.

### 2.2. Data Collection

Upon admission into the ICUs, the following items were recorded for each patient: source of patients, age, gender, chief complaints for admission, symptoms, temperature, Acute Physiology and Chronic Health Evaluation (APACHE) II scores [21], SOFA scores [19], mechanical ventilation, continuous renal replacement treatment (CRRT), etiological factors, pathogens, and underlying diseases. Within 24 h (first day of study) after ICU admission and in the morning of days 3, 5, 7, 10, and 14, intravenous blood samples were obtained and centrifuged at 3,000 rpm for 15 min. The supernatants were transferred to Eppendorf tubes and stored at −80°C.

### 2.3. Assays

All the specimens were renumbered before the experiment. We ensured that each step was blind to researchers. sTREM-1 was determined with a double antibody sandwich enzyme-linked immunosorbent assay (ELISA) (Quantikine Human TREM-1 Immunoassay ELISA Kit, R & D Systems, Minneapolis, Minnesota, the United States, product number DTRM10B); sCD163 was determined with a double antibody sandwich ELISA (soluble CD163 ELISA assay for the measurement of macrophage and monocyte activation, IQ Products, The Netherlands, product number IPQ-383); CRP was determined by scattering turbidimetry (CardioPhase hsCRP, Siemens, Germany); and PCT, and by enzyme-linked fluorescence analysis (ELFA, VIDAS BRAHMS PCT kit, bioMerieux SA, France). ELISA was performed in duplicate and all the other assays were done in strict accordance with the manufacturers' instructions.

### 2.4. Statistical Analysis

Results for continuous variables with normal distributions, including age, temperature, WBC counts, serum CRP, APACHE II scores, and SOFA scores, are given as means ± standard deviations (SDs). Student's *t*-test was performed to compare means between two groups. Analysis of variance (ANOVA) was made to compare means among multiple groups and interpreted based on post hoc comparisons. Results from continuous, abnormally distributed variables, including serum sTREM-1, sCD163, and PCT, are given as medians (interquartile ranges) and were compared by means of nonparametric tests. Results for qualitative variables, such as source of patients, gender, mechanical ventilation (MV), CRRT, etiological factors, pathogens, predisposing factors, and the mortality rate, were denoted as percentages and compared across groups by means of a Chi-square test. Logistic regression analysis was carried out to estimate the odds ratio (OR) and the 95% confidence interval (CI). Stepwise and forward selection procedures were introduced to select iteratively variables possibly related to sepsis. To be entered into this model, a *P* < 0.05 from logistic regression model was required. Factors related to survival were explored through Cox regression and calculating the hazard ratios. The AUC (areas under receiver operating characteristic curves) method was employed to evaluate how well the model works in distinguishing sepsis from SIRS, and severe sepsis, and in predicting prognosis. For statistical analysis, SPSS 16.0 (SPSS, Chicago, Illinois, USA) was used, and a two-tailed *P* < 0.05 was considered significant.

## 3. Results

### 3.1. Subjects Descriptions

As this study focused on the dynamics of different biomarkers, patients who died within 24 h after being admitted into the ICU, refused to participate in the study, or quit further treatment on their own within 14 days failed the requirements for continuous observation. A total of 130 patients, selected out of 377 in accordance with relevant criteria, were formally included in this study (see Supplementary Figure 1 in Supplementary Material available online at http://dx.doi.org/10.1155/2013/969875). 30 critical patients with two or more SIRS signs and negative pathologic examination results, who were from the SICU within 24 hours after aseptic surgery, were also selected as SIRS control group in the study. These patients had received a general examination to exclude infection within 24 hours before surgery. In light of the sepsis guidelines, the 100 sepsis patients were further divided into a sepsis subgroup (36 cases), a severe sepsis subgroup (35 cases), and a septic shock subgroup (29 cases). Baseline data at admission into ICU are shown in Table 1. APACHE II and SOFA scores go markedly from high to low in the following order: septic shock > severe sepsis > sepsis subgroup (*P* < 0.001). More septic shock patients are in need of mechanical ventilation than sepsis patients (*P* = 0.026). With a 28-day survival as a criterion, mortality rate for the septic shock subgroup is the highest, followed by the severe sepsis subgroup, and the sepsis subgroup ranks the lowest (*P* < 0.001). Statistically, there are no remarkable differences in terms of age, gender, temperature, etiological factors (excluding catheter-related bloodstream infection), pathogens, or accompanying underlying diseases (excluding coronary heart disease and the immunosuppressed condition) between groups. Additionally, it should be explained that some patients had multiple pathogens and/or infection of multiple sites.

### 3.2. sTREM-1, sCD163, PCT, CRP, and WBC Counts: Values for Early Sepsis Diagnosis

**Figure 1:**
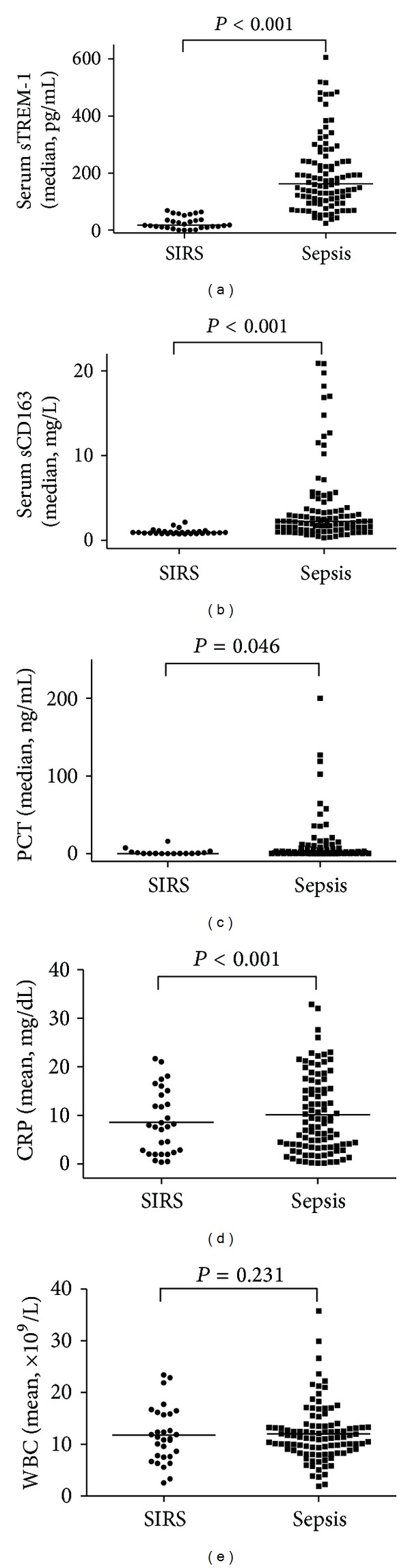
Serum sTREM-1 (a), serum sCD163 (b), PCT (c), CRP (d), and WBC (e) according to the sepsis diagnosis criteria. (SIRS (*n* = 30) versus sepsis (*n* = 100)). The dots denote individual values, and the bars indicate medians or means. Serum sTREM-1, serum sCD163, PCT, and CRP levels come out as 180.92 (150.44) pg/mL versus 29.41 (20.77) pg/mL, *P* < 0.001; 2.22 (2.36) mg/dL versus 0.88 (0.23) mg/dL, *P* < 0.001; 1.65 (10.1) ng/mL versus 0.35 (1.58) ng/mL, *P* = 0.046; 11.76 ± 8.09 mg/dL versus 5.65 ± 4.27 mg/dL, *P* < 0.001, respectively. But a comparison of WBC level between the two groups is devoid of such significance (12.19 ± 6.01 × 10^9^/L versus 11.27 ± 2.54 × 10^9^/L, *P* = 0.231).

On the first day of ICU enrollment, the sepsis group exhibited a higher level in serum sTREM-1, serum sCD163, PCT, and CRP than the SIRS group (Figure 1). Univariate analysis was made to assess possible risk factors to sepsis. The variables taken into account included serum sTREM-1, sCD163, CRP, PCT, WBC counts, and APACHE II score (Table 2). Four variables, sTREM-1, sCD163, CRP, and APACHE II score, were further selected for multivariate regression (*P* < 0.001). Finally, only serum sTREM-1 entered the multivariable regression equation, with OR = 1.089 (95% CI 1.045–1.136, *P* < 0.001). The receiver operating characteristic (ROC) curves were used to calculate serum sTREM-1's performance in sepsis diagnosis (Supplemental Figure 2). AUC turned out to be 0.978 (95% CI 0.958–0.997). With a cut-off point of 64.4 pg/mL for sTREM-1, sensitivity came out as 0.91; specificity, 0.896; PPV, 0.989, and NPV, 0.621.

### 3.3. Serum sTREM-1 sCD163, PCT, CRP, and WBC: Values for Severity Assessment of Sepsis

**Figure 2:**
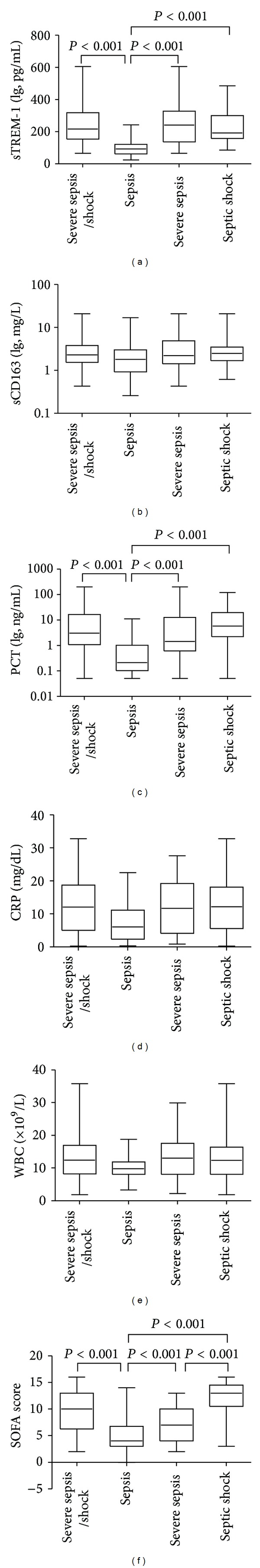
Serum sTREM-1 (a), serum sCD163 (b), PCT (c), CRP (d), WBC (e), and SOFA score (f) on the ICU admission day when sepsis (36 cases), severe sepsis (35 cases), and septic shock (29cases) occurred. Severe sepsis/shock group is defined as a state which represents sepsis severity, including severe sepsis and septic shock (64 cases). *y*-axis of sCD163 and PCT is labeled as logarithmic.

**Figure 3:**
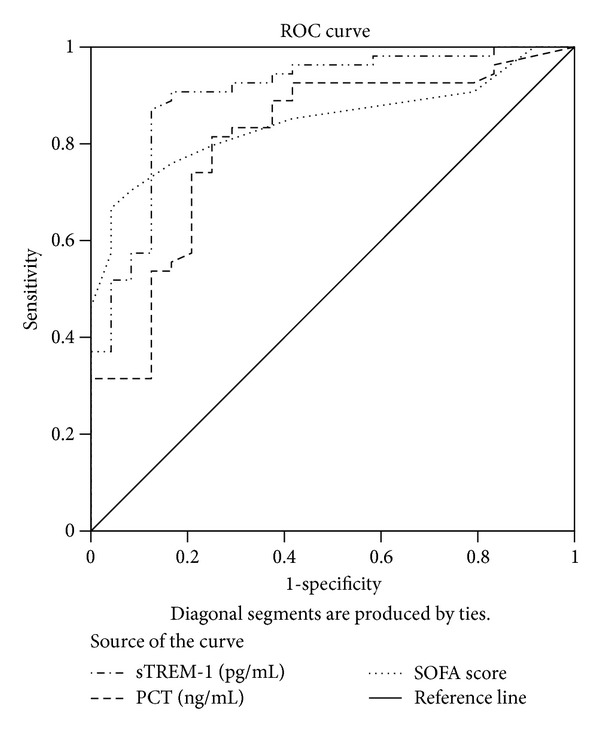
ROC curves for serum sTREM-1 and PCT levels for severe sepsis (severity of sepsis). AUC demonstrates that serum sTREM-1 measures 0.9 (95% CI 0.823–0.977), serum PCT measures 0.806 (95% CI 0.7–0.913), and SOFA score measures 0.846 (95% CI 0.716–0.931). With 136.82 pg/mL as the cut-off point for sTREM-1, sensitivity measures 0.87 and specificity 0.88; with 0.83 ng/mL as the cut-off point for PCT, sensitivity measures 0.82 and specificity 0.75; with 8.5 as the cut-off point for SOFA score, sensitivity measures 0.67 and specificity 0.96.

Figure 2 illustrates a pairwise comparison over serum sTREM-1, WBC counts, serum CRP, serum PCT, and SOFA score between the sepsis, severe sepsis, and septic shock groups, made on the first day of enrollment. It turned out that the sepsis group scored the lowest in sTREM-1, PCT, and SOFA score, which was of statistical significance, compared with any other group. As for the severe sepsis group and the septic shock group, statistically, of all the indicators, only the disparity in the SOFA score between the two deserved attention. So we combined severe sepsis and septic shock groups into a severe sepsis/shock group to express the seriousness of the sepsis condition. Higher serum sTREM-1, PCT level, and SOFA score in severe sepsis/shock group (*P* < 0.05). Although severe sepsis/shock group had higher sCD163, CRP, and WBC levels, a comparison of such indicators across groups is devoid of such significance. ROCs for serum sTREM-1, PCT, and SOFA score illustrate severe sepsis/shock group, which reflect sepsis severity; see Figure 3. 

### 3.4. Serum sTREM-1 sCD163, PCT, CRP, and WBC Counts: Values for Dynamic Assessment of Sepsis Prognosis

**Figure 4:**
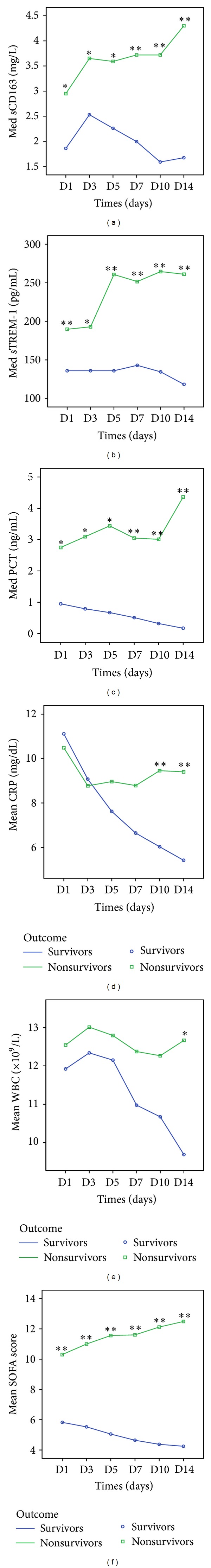
Serum sCD163 level (a), Serum sTREM-1 level (b), PCT level (c), CRP level (d), and WBC counts (e) measured over 14 days in patients diagnosed with sepsis, based on 28-day survival. The differences in serum sCD163, sTREM-1, and PCT levels at these 6 different time points were statistically significant, with the nonsurvivors group having higher values at all time points and also showing a higher CRP level on days 10 and 14, which were also statistically significant. WBC counts in the nonsurvivors group were also higher than those of the survivors group, but only one time point (day 14) registered difference statistically significant. Survivors = 57; nonsurvivors = 43; *P < 0.05; **P < 0.01.

Based on the 28-day survival, sepsis patients were also divided into a survivors' group and a nonsurvivors' group.  Figure 4 compares these two groups in terms of dynamic changes in serum sTREM-1, sCD163, WBC counts, serum CRP, and serum PCT levels. The curves show that the nonsurvivors' group had higher serum sTREM-1, sCD163, WBC counts, serum PCT levels, and SOFA score during this period of time. For nonsurvivors, their serum sTREM-1, sCD163, serum PCT levels, and SOFA score increased with the passage of time, while their WBC counts and serum CRP levels tended to decline. In contrast, all indicators of the survivors' group revealed a tendency to decline. The serum sCD163, sTREM-1, and PCT levels of nonsurvivors were higher than survivors' at these 6 different time points (*P* < 0.05).

**Figure 5:**
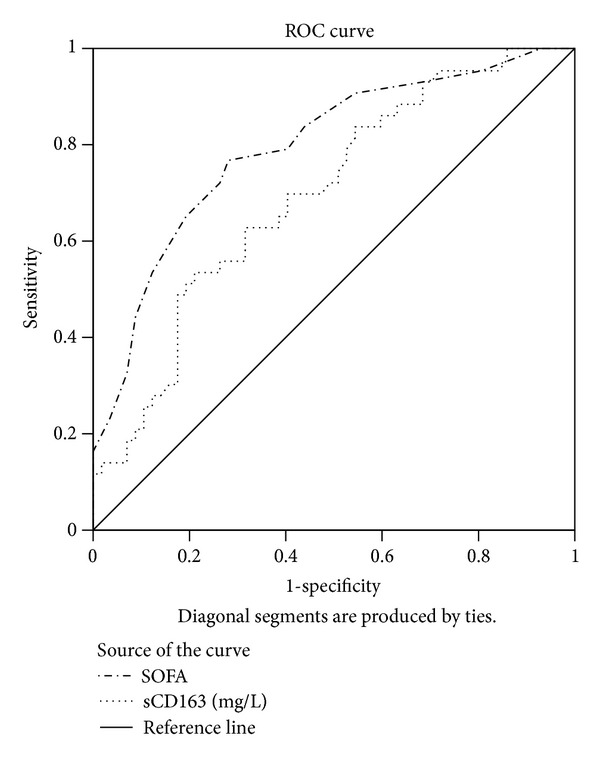
ROC curves for serum sCD163 and SOFA score for sepsis prognosis. AUC demonstrates that serum sCD163 measures 0.696 (95% CI 0.593–0.799) and SOFA score measures 0.794 (95% CI 0.705–0.833). With 2.84 mg/L as the cut-off point for sCD163, sensitivity measures 0.535 and specificity 0.789, positive predictive value (PPV) 0.657, and negative predictive value (NPV) 0.692; with 7.5 as the cut-off point for SOFA score, sensitivity measures 0.767, specificity 0.719, PPV 0.673, and NPV 0.804.

Cox regression was employed to analyze the survival time of sepsis patients, as well as the factors affecting survival. The variables taken into account included sex, age, temperature, serum sTREM-1, sCD163, WBC, CRP, PCT, APACHE II score, SOFA score, use of life support technology (e.g., MV and CRRT), etiological factors, pathogens, and predisposing factors. Within 24 h after the ICU admission, the indicators previously mentioned were derived from the patients. Finally, only sCD163 and SOFA entered the regression equation. For the former, the regression coefficient = 0.09, hazard ratios = 1.09 (95% CI 1.035–1.154, *P* < 0.001), whereas for the latter, the regression coefficient = 0.2, hazard ratios = 1.23 (95% CI 1.126–1.335, *P* < 0.001). The ROC curve denoting these two survival-affecting parameters was drawn on the ICU admission day in order to predict prognosis (Figure 5). 

## 4. Discussions

Currently, the exact role of biomarkers in the assessment of septic patients remains obscure [22]. Although 178 related sepsis biomarkers have been identified, it is still controversial which is reliable for sepsis diagnosis [23]. In particular, PCT and CRP, which have been most widely used in clinical treatment, have limited ability to distinguish sepsis from other inflammatory conditions or to predict outcome. Therefore, the exploration and discovery of sepsis biomarkers still should be paid attention to.

Soluble sTREM-1 is identified as a marker of microbial infection by many studies [24–27]. The upregulation of CD163 at the occurrence of sepsis, caused by activating the waterfall effect from the secretion of anti-inflammatory cytokines, helps scavenge hemoglobin and reduce its oxidative impairment to the body [28, 29]. CD163 is also innately immune and bacterial flora identifying [30]. We found the same phenomenon that, on the admission day, the sepsis group exhibited a higher level of sTREM-1, sCD163, PCT, and CRP than the SIRS group. In addition, the disparity was of statistical significance (*P* < 0.05). That reveals that the indicators previously mentioned are all applicable to early sepsis diagnosis. Multivariate logistic regression displays that serum sTREM-1 is the only risk indicator for sepsis diagnosis. The ROC area for serum sTREM-1 came out as 0.978 (95% CI 0.958–0.997) and both sensitivity and specificity, around 0.9. The diagnostic value of sTREM-1 is obviously higher than sCD163, CRP, and PCT. That is to say, serum sTREM-1 may prove a better indicator for the sepsis diagnosis.

The serum sTREM-1 and PCT levels as well as SOFA score can play a role in severity assessment of sepsis. The value of the three indicators from the severe sepsis group, the septic shock group, and the severe sepsis/shock group all exceeded that from the sepsis group (*P* < 0.01). In a comparison between the severe sepsis group and the septic shock group, only the sofa score possesses a certain significance. To sum up, the sTREM-1 and PCT level and the SOFA score are of diagnostic value for sepsis severity. In addition, sTREM-1 has the highest efficiency, with a ROC area of 0.9; sensitivity turned out to be 0.87, and specificity, 0.88 with 136.82 as the cut-off point for severe sepsis diagnosis. What is interesting is that the severity assessment value of sCD163 is limited. It might be assumed that the expression of sCD163 on the surface of macrophage membrane is regulated by more than one factor. Studies show that interleukin-6 (IL-6) and interleukin-10 (IL-10) stimulate, whereas lipopolysaccharide (LPS) and interferon-*γ* (IFN-*γ*) contain, the expression of CD163 molecules on the surface of macrophage membrane [31]. In the meantime, only with the involvement of metalloprotease, LPS, and at least one inflammatory medium, could a drop of CD163 into sCDl63 be possible, by means of activating Toll-like receptors [32]. Therefore, the state and expression of CD163 are governed by the internal environment of the human body. It could also be assumed that the high expression of sCD163 is related to the positive feedback of inflammation. That is to say, the expressive volume of sCD163 is limited right after inflammatory responses are activated. 

Dynamic changes in serum sTREM-1 may prove helpful for prognostic assessment [33, 34]. More than one study reports that sCD163 is more valuable for earlier prognostic assessment [35, 36]. We found that, seen from the dynamic tendency of the curve denoting sepsis prognosis, the differences in serum sTREM-1, CRP, and PCT level as well as in SOFA score at these six different time points were statistically significant, with the nonsurvivors' group having higher values all the time, and showing a higher CRP and WBC level even at the final stage, which was also statistically significant. For the nonsurvivors, sTREM-1, sCD163, and PCT level as well as SOFA score went up with the passage of time, whereas for the survivors, these indicators tended to decline. This demonstrates that sTREM-1, sCD163, and PCT, as well as SOFA score, have their value for clinical application in dynamic assessment of sepsis prognosis. Relevant factors affecting survival within the first 24 h of ICU stay were analyzed and sorted out using Cox regression. It turned out that only the sCD163 level and SOFA score entered the equation and served as the independent risk factors affecting survival. Further analysis was made of sCD163 level and SOFA score by means of ROC curve to determine the cut-off point. Therefore, these two factors are likely to function as an index for reference in terms of early prognosis assessment. sTREM-1, however, did not work well in prognostic assessment as a risk factor. The reason may lie in the fact that it is protective to inflammation at the initial stage. At the onset, sTREM-1 may combine with membrane-bound TREM-1 by competitive, ligand binding, or with DAP-12, an inhibitive receptor, by specific binding, thus containing human body's excessive responses to inflammation. With the development of the disease and the inflammatory cascade amplification, the above combinations reach a saturation point. sTREM-1 accumulates and is then released into the blood in large quantities. For these reasons, sTREM-1 level at the initial stage may be fairly low and insensitive in early prognostic assessment and could only play a better role in later stage, dynamic prognostic assessment. 

The present study, however, has its own limitations. First, central tendency values were used to describe the dynamics of different biomarkers. It is very helpful to draw conclusions about decisions for individual patients and their values. However, owing to the limitations of our sample size, prospective clinical studies are still wanted to provide further proof for the clinical diagnostic value of these biomarkers. Second, the internal environment of the human body is an important contributor to the expression of the markers identified in this study. The internal environment can be influenced by clinical care (drugs, timing, dose, mechanical ventilation technique, CRRT, etc.). Clinical care environment may also have impact on the responses of their patients and therefore on their measurements. We cannot negate the impact of such factors. Third, the purpose of this study is to observe the dynamic changes of the various indicators. For this, it precludes a considerable portion of candidates according to the exclusion criteria. At the same time, the SIRS patients, without both prior surgery and 24 h postsurgery infections were selected as control group. This study also does not rule out impact of the specific conditions on the enrollees.

In summary, it may be concluded from the study that sTREM-1 is more ideal than PCT and CRP for early sepsis diagnosis and severity assessment and constitutes an independent risk diagnostic parameter. sCD163 and SOFA scores possess positive clinical values in dynamic, prognostic assessment and function as independent, survival-affecting risk factors. Future studies with larger subject populations and with attention to the clinical care environment are expected to define the application of these parameters in clinical decision making.

## Supplementary Material

Trail profile for patients enrolled in this study.

## Figures and Tables

**Table 1:** Clinical and biological data at admission in ICU according to the diagnosis of sepsis and its severity.

Characteristics	All SIRS	All sepsis	*P* value	Sepsis	Severe sepsis	Septic shock	*P* value
	*N* = 30	*N* = 100		*N* = 36	*N* = 35	*N* = 29	
Age (years)	52.2 ± 20.4	58.9 ± 19.5	0.105	57.2 ± 19.9	55.3 ± 18.5	65.4 ± 19.1	0.094
Gender (*n*, %)			0.091				0.753
Male	15 (50)	67 (67)		23 (63.9)	23 (65.7)	21 (72.4)	
Female	15 (50)	33 (33)		13 (36.1)	12 (34.3)	8 (27.6)	
Temperature (°C)	37.2 ± 0.6	37.8 ± 1.3	<0.001	37.9 ± 1.1	38.0 ± 1.1	37.4 ± 1.6	0.112
APACHE II score	11.0 ± 7.0	13.4 ± 6.1	<0.001	12.7 ± 6.4	18.4 ± 7.2	23.1 ± 5.4	<0.001
SOFA score	—	7.8 ± 4.4	—	4.7 ± 3.0	7.3 ± 3.6	12.1 ± 3.0	<0.001
MV (*n*, %)	23 (76.7)	80 (80)	0.693	24 (66.7)	29 (82.9)	27 (93.1)	0.026
CRRT (*n*, %)	1 (3.3)	22 (22)	0.019	7 (19.4)	8 (22.9)	7 (24.1)	0.892
Possible etiological factors (*n*, %)							
Pulmonary infection	—	83 (83)	—	30 (83.3)	32 (91.4)	21 (74.2)	0.129
Abdominal infection	—	18 (18)	—	6 (16.7)	3 (8.6)	9 (31)	0.064
Urinary tract infection	—	24 (24)	—	11 (30.6)	6 (17.1)	7 (24.1)	0.417
Trauma/postoperative infection	—	31 (31)	—	12 (33.3)	10 (28.6)	9 (31)	0.154
Bacteremia	—	23 (23)	—	12 (33.3)	7 (20)	4 (13.8)	0.091
Catheter-related infections	—	13 (13)	—	10 (27.8)	1 (2.9)	2 (6.9)	0.004
Others	—	4 (4)	—	0 (0)	1 (2.9)	3 (10.3)	0.074
Pathogens detected							
Gram-positive bacteria	—	37 (37)	—	15 (41.7)	8 (22.9)	14 (48.3)	0.085
Gram-negative bacteria	—	81 (81)	—	31 (86.1)	28 (80.0)	22 (75.9)	0.568
Fungi	—	62 (62)	—	25 (69.4)	21 (60.0)	16 (55.2)	0.471
Predisposing factors (*n*, %)							
Hypertension	9 (30)	41 (41)	0.277	13 (36.1)	18 (51.4)	10 (34.5)	0.295
Diabetes	2 (6.7)	16 (16)	0.319	3 (8.3)	7 (20)	6 (20.7)	0.261
COPD	0 (0)	14 (14)	0.067	6 (16.7)	3 (8.6)	5 (17.2)	0.493
Coronary heart disease	3 (10)	17 (17)	0.54	4 (11.1)	3 (8.6)	10 (34.5)	0.016
Immunosuppressed condition	0 (0)	11 (11)	0.127	3 (8.3)	8 (22.9)	0 (0)	0.004
Nervous system disease	0 (0)	12 (12)	0.103	5 (13.9)	2 (5.7)	5 (17.2)	0.304
CKD	1 (3.3)	8 (8)	0.636	4 (11.1)	2 (5.7)	2 (6.9)	0.687
28-day mortality rate (*n*, %)	2 (5.0)	43 (43.0)	*P* < 0.001	6 (16.7)	17 (48.6)	20 (69)	*P* < 0.001

Quantitative data of normal distribution are presented as mean ± SD. Quantitative data of nonnormal distribution are presented as median (interquartile range). Qualitative data are presented as *n* (%).

RICU: respiratory intensive care unit; SICU: surgical intensive care unit; EICU: emergency intensive care unit; MV: mechanical ventilation; CRRT: continuous renal replacement treatment; APACHE II score: acute physiologic assessment and chronic health evaluation II scores; SOFA score: sequential organ failure assessment scores; CKD: chronic kidney disease.

**Table 2:** Univariate analysis of dichotomous variables for the purpose of distinguishing sepsis from SIRS.

Variable	*β*	S.E.	Wald	*P *	OR	95% C.I. for OR	
						Lower	Upper
sTREM-1	0.08	0.02	17.65	<0.001	1.09	1.04	1.13
sCD163	2.02	0.51	15.76	<0.001	7.55	2.78	20.49
WBC	0.03	0.04	0.66	0.42	1.03	0.95	1.12
CRP	0.12	0.04	8.76	<0.001	1.12	1.04	1.22
PCT	0.08	0.07	1.57	0.21	1.08	0.95	1.23
APACHE II	0.09	0.03	10.18	<0.001	1.10	1.04	1.16
