# Supplementary material for: Diagnostic Value of Dynamics Serum sCD163, sTREM-1, PCT, and CRP in Differentiating Sepsis, Severity Assessment, and Prognostic Prediction
Source: Mediators Inflamm. 2013 Jul 1;2013:969875. doi: 10.1155/2013/969875 (PMC3713373; doi:10.1155/2013/969875)

Supplement Figure Legends

Supplement Figure 1 Trail profile for patients enrolled in our study

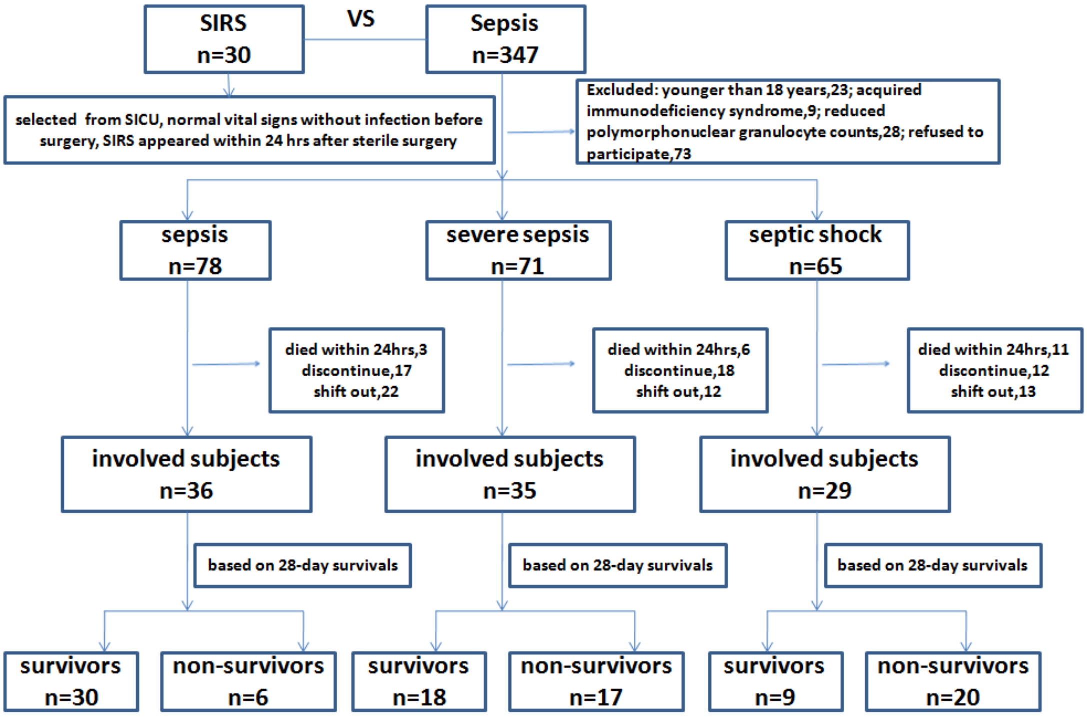

Supplement Figure 2 ROC curves for serum sTREM-1 levels for distinguishing sepsis from SIRS.

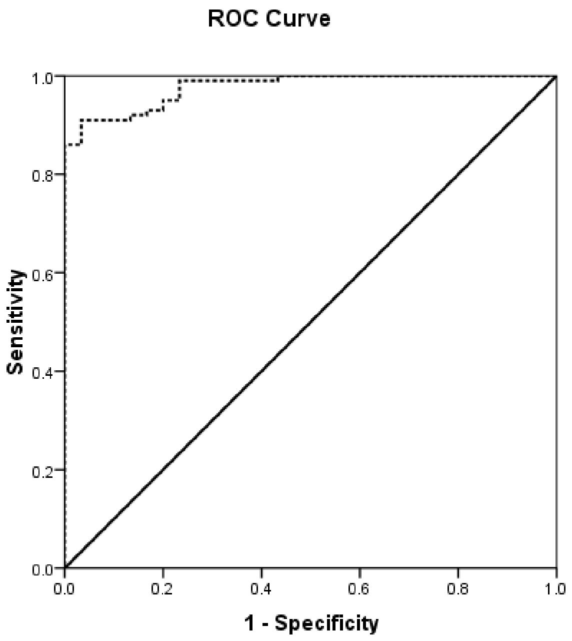

Supplement: Supplementary file 1 — Trail profile for patients enrolled in this study. [file 969875.f1.pdf]
